# Outcomes of neonates with tracheostomy secondary to bronchopulmonary dysplasia

**DOI:** 10.1186/s12887-020-02324-1

**Published:** 2020-09-01

**Authors:** Kirtikumar Upadhyay, Dario Antonio Vallarino, Ajay J. Talati

**Affiliations:** 1 Division of Neonatal Medicine, Department of Pediatrics, University of Tennessee Health Science Center, 853 Jefferson Avenue, Suite E#201, Memphis, TN 38103 USA; 2 Department of Pediatrics, University of Washington, Seattle, USA; 3 Division of Neonatology, Kids Medical Services, Miami, FL USA; 4 Department of OB/GYN, University of Tennessee Health Science Center, Memphis, TN USA

## Abstract

**Background:**

Bronchopulmonary dysplasia (BPD) is a disease that can affect preterm neonates. Infants with severe BPD may develop pulmonary hypertension (PHN) and may require chronic mechanical ventilation with tracheostomy. The outcomes of these infants have not been studied well. We proposed to review survival and outcomes of infants requiring tracheostomy secondary to severe BPD in our NICU at 24 months.

**Methods:**

We reviewed infants’ charts who were diagnosed with BPD that underwent tracheostomy from January 2011 to May 2016 at our children’s hospital NICU. Data were recorded from hospital stay as well as from follow up clinics. Institutional review board approval was obtained prior to beginning of study.

**Results:**

Forty-one babies (37 during initial hospitalization and 4 subsequently) requiring tracheostomy were identified from our database. Median gestational age at birth was 26 weeks (25–27 IQR), mean birthweight of 731 g (±245 SD) and 32% were small for gestational age (SGA). Median age of tracheostomy placement was 168 days (108–197 IQR), and median PMA 48 wks (40–56 IQR). 26% of infants requiring tracheostomy also had subglottic stenosis along with BPD. 34 infants (83%) survived to discharge from NICU. 66% (27/41) of our patients had a composite outcome of death, ventilator dependency and/or poor neurodevelopmental outcome at 2 years. We found that a higher respiratory severity score at the time of tracheostomy placement and later post-menstrual age at admission to level IV NICU was associated with a worse outcome. Small for gestational age infants were found to have worse outcomes as well. 41% (13/32) of infants had more than 3 hospital admissions after discharge.

**Conclusions:**

In our cohort about 80% of infants with severe BPD and tracheostomy survived to discharge with need for prolonged home ventilation in more than half of the survivors. Later postmenstrual age at admission to level 4 NICU was associated with a worse outcome. Our retrospective data may be inadequate to determine the causal relationship between postmenstrual age at admission and outcome. These patients continue to have high morbidity and recurrent hospitalizations.

## Background

With advanced technology in health care, survival of extremely low birth infants has been increasing and consequently morbidity associated with prematurity like bronchopulmonary dysplasia (BPD) is on a rise [1–6]. Premature infants with BPD are at high risk of mortality, prolonged neonatal intensive care unit (NICU) stay, and significant medical support needs following NICU discharge [7, 8]. Concurrent with changes in pulmonary management, the incidence of severe BPD has decreased in recent years [9]; however, there remain a subset of infants with BPD whose illness requires chronic, intensive pulmonary management [10]. Currently, no national guidelines or standardized care models are available for this population. In addition, some of these infants require placement of a tracheostomy to deliver long-term mechanical ventilation [11].

Infants with severe BPD are generally referred to tertiary NICUs at children’s hospitals for higher level of care [12, 13]. The timings of referral to regional NICU [14] and placement of tracheostomy [15] is not uniform across many centers in US [16, 17]. Decisions about whether and when to place a tracheostomy in preterm infants who require prolonged ventilation are challenging due to lack of objective data. Clinicians face significant challenges in counselling these families of infants with severe BPD; which in turn creates more anxiety among parents and subsequently may lead to delay in tracheostomy placement. We wanted to review characteristics and outcomes of infants requiring tracheostomy secondary to severe BPD in our level IV NICU. Our objective was to identify the risk factors associated with poor outcomes of these infants. Such information could help inform clinicians when making decisions with families about the timing of tracheostomy placement and planning for the care of these children throughout early childhood.

## Methods

Our neonatal intensive care unit at Le Bonheur Children Hospital is a tertiary care referral center and an urban, inner city unit. It serves many patients from underserved African American communities who were out born and were transferred to our NICU for multi-specialty involvement with complex needs. Our center is a referral center for west Tennessee and adjacent counties of Arkansas and Mississippi. Preterm infants with severe BPD are reviewed and evaluated by multidisciplinary team consisting of neonatology, pediatric pulmonology, pediatric cardiology, pediatric ENT specialist, pediatric anesthetist, nursing teams and physical/occupational therapists. For infants developing severe BPD, our approach include aggressive monitoring (q4weeks echocardiogram) and management of pulmonary hypertension using pulmonary vasodilators such as sildenafil and bosentan, permissive hypercapnia (PCO2 55–65 mm of Hg) and higher target oxygen saturations (92–98%). For infants over 36 weeks of corrected gestational age, our ventilation strategies included high tidal volume (8–10 ml/kg), low vent rate (20/min) and high inspiratory time (Ti) > 0.5. We aimed to provide caloric dense feedings with a daily goal of 125-130 kcal/kg/day and 4.5 g/kg/day of enteral protein to these infants for optimal somatic, linear and lung growth. The timing of placement of tracheostomy in severe BPD infants was dependent on inputs received from interdisciplinary teams. Generally, chronic ventilator dependent status after 44 weeks of corrected gestational age and strong family support/desire for long-term care led to tracheostomy placement. All our NICU graduates were closely followed up by pulmonologists, developmental pediatricians and psychologists. Developmental assessment was performed as early as 6 months of age and early intervention therapy was offered whenever deemed necessary. The study population derived from the entire population of infants admitted to our NICU from January 2011 to May 2016. Premature infants with severe BPD who underwent tracheostomy placement during this period were identified from our database. We reviewed these infants’ charts to confirm the eligibility and data collection. Inclusion criteria were preterm infants less than 34 weeks of gestational age admitted in our level 4 NICU during study period. Infants with complex congenital anomalies, lethal anomalies, trisomy 13 or 18 were excluded.

Data were recorded from hospital stay as well as from follow up clinics. Data were collected for infant and maternal demographics and clinical characteristics. Long term follow-up was assessed at 24 months by noting survival, respiratory status, need for mechanical ventilation and developmental status. Definitions for maternal and infant characteristics were predetermined i.e. gestational age (GA) was determined as the best obstetric estimate or postnatal assessment. Small for GA was defined as BW of <10th percentile using Fenton growth charts. BPD was defined according to the National Institute of Child Health and Development (NICHD) workshop definition [18]; Mild BPD was defined as a need for supplemental oxygen (O_2_) for ≥28 days but not at 36 weeks’ postmenstrual age (PMA) or discharge, moderate BPD as O_2_ for ≥28 days plus treatment with < 30% O_2_ at 36 weeks’ PMA, and severe BPD as O_2_ for ≥28 days plus ≥30% O_2_ and/or positive pressure at 36 weeks’ PMA. Respiratory Severity Score (RSS) was calculated as mean airway pressure (MAP) multiplies by FiO2. RSS data were collected on admission to our institution and immediately prior to tracheostomy placement. Pulmonary hypertension (PHN) was diagnosed based on echocardiogram criteria by Pediatric Cardiologists and if it required medical management. After discharge, our pulmonology team followed these infants at home and utilized data such as pulse oximeter, end tidal CO2 measurement and polysomnography for weaning off the home ventilator. Composite outcome of death or mechanical ventilator dependence and/or poor neurodevelopmental outcome (poor neurodevelopmental outcome defined as MDI and PDI < 70 by Bayley’s scale of Infant development or GMFCS ≥ level III) at 24 months of corrected age. The study was approved by the Institutional Review Board at the University of Tennessee Health Science Center. Data were analyzed by descriptive statistics using mean and median. Groups were analyzed using t-test. Categorical variables were compared with chi-square analysis. We used the forward stepwise logistic regression analysis for our dataset. This involved starting with no variable in the model, followed by adding a variable whose inclusion gave the most statistically significant outcome and repeating the process until none improved the model to a statistically significant extent. If a nonsignificant variable was found then it was removed from the model.

## Results

Out of 2435 total admissions in NICU from January 2011 to May 2016; 41 infants (1.68%) required tracheostomy due to prolonged mechanical ventilator need because of BPD. Median gestational age at birth was 26 weeks (23–27 IQR) and mean birthweight was 731(±245) gm. Total 66% (27/41) of infants were born via c- section and 73% (29/41) of infants received antenatal steroids. All infants received surfactant (median 2 doses) and at least one course of postnatal steroids. Almost all infants (98%) were referred from other level III NICUs. Median peak FiO2 during the first 30 days was 0.40 (0.35–0.44 IQR), at 36 weeks post-menstrual age (PMA) 0.30 (0.25–0.4 IQR), and at time of tracheostomy placement 0.35 (0.32–0.45 IQR). PHN was diagnosed in 61% (25/41) of our cases which was associated with 36% (9/25) mortality. 88% (22/25) of infants with pulmonary hypertension received sildenafil. Median age of tracheostomy placement was 168 days (108–197 IQR), and median PMA 48 wks (40–56 IQR). 26% of infants requiring tracheostomy also had subglottic stenosis along with BPD. G-tube was placed on all surviving infants- 83% (34/41), while 17% (7/41) died prior to discharge from NICU and 66% (27/41) of our patients had a composite outcome of death, home mechanical ventilation need and/or poor neurodevelopmental outcome by 2 years of corrected age. As shown in Table 1, we found that infants who were transferred to our NICU at a later post-menstrual age had worse outcome compared to the infants who were transferred earlier after birth. (35 vs. 39 wks, *p*-value 0.03). Small for gestational age infants had poor outcomes as well. Higher RSS prior to tracheostomy placement was associated with poor outcomes. The other characteristics of infants with good and poor outcomes have been described in Table 1. 41% (14/34) of infants had more than 3 hospital admissions after discharge from NICU.

**Table 1:** The characteristics of infants with BPD who received tracheostomy

Variable	Poor Outcome (*n* = 27)	Good outcome (*n* = 14)	*P* value
Gestational Age (wks) Mean, SD	26.5 ± 1.8	26 ± 2.3	0.44
Post-conceptional age at admission (wks) Mean, SD	39.1 ± 7.5	34.6 ± 5.4*	0.05
Male (%)	19/27 (70%)	9/14 (64%)	0.69
Birthweight (kg) Mean, SD	0.68 ± 0.17	0.83 ± 0.34	0.06
Small for gestational age	12/27	2/14	0.11
FiO2 at 36 wks CGA Mean, SD	0.40 ± 0.17	0.30 ± 0.08*	0.04
RSS prior to tracheostomy Mean, SD	4.8 ± 1.8	3.6 ± 1.4*	0.03
Post-conceptional age at tracheostomy (wks) Mean, SD	50.9 ± 8.9	49.6 ± 14.6	0.72
Pulmonary Hypertension (%)	19/27 (70%)	6/14 (43%)*	0.049
Sepsis	14/27 (51%)	6/14 (43%)	0.58
Mode of delivery	18/27 (66%)	9/14 (64%)	0.87
Postnatal steroids	27/27 (100%)	14/14 (100%)	0.65
Antenatal steroids	18/27 (66%)	11/14 (78%)*	0.42

(* *p* value ≤ .05)

Furthermore, forward stepwise logistic regression data suggest that poor outcomes at 2 years of corrected age in infants with severe BPD and tracheostomy was associated with SGA status at birth, pulmonary hypertension, RSS at the time of tracheostomy placement and PMA at the time of admission to our institution (Table 2). The surviving infants were followed for 2 years of corrected age. More than half of infants in our cohort were found to be ventilator dependent at 2 years of age and of which 61% had poor neurodevelopmental outcome at 24 months of corrected age (Table 3).

**Table 2:** Forward step logistic regression analysis for risk factors associated with poor outcomes in infants with severe BPD requiring tracheostomy

Infant characteristics	Odds Ratio Estimate	
	Point estimate	95% Wald Confidence Interval
Post conceptional age at admission (continuous)	2.251*	1.921–2.699
Higher RSS prior to tracheostomy placement	1.985*	1.635–3.438
Small for gestational age	3.577*	1.123–11.391
Antenatal steroids	1.195	0.570–2.503
Pulmonary hypertension	3.972*	1.683–9.373

*p value < 0.05

**Table 3:** Outcomes at 24 months in surviving infants. Data are not mutually exclusive

Outcomes at 24 months (corrected age)	*n* = 34
Death after discharge from NICU	2 (6%)
Ventilator dependent	18 (53%)
MDI and PDI < 70 or GMFCS ≥ level III	11 (32%)
Hospital readmission (more than 3 times)	14 (41%)

*MDI* mental development index, *PDI* psychomotor development index, *GMFCS* gross motor function classification system

## Discussion

Parents experience overwhelming anxiety and stress when discussing tracheostomy placement and they often worry about “how long my child will be dependent on mechanical ventilator for his/her breathing?“ [19].In a single center cohort of infants with severe BPD and tracheostomy, our data demonstrated that median post menstrual age for placement of tracheostomy was 48 weeks. This is consistent with other reports in the literature [11]. The timing of decannulation and independence from ventilator is an extremely important milestone for infants with severe BPD [20]. Our study showed that 2/3rd of infants requiring tracheostomy were vent dependent even at 2 years of age. Our data showed that higher respiratory severity score at the time of tracheostomy placement and later gestational age was associated with poor outcomes at 24 months of corrected gestational age. These are our data based on institutional practice, but there is no national consensus on timing of tracheostomy placement for BPD. We want to caution the readers that these findings may not be applicable to all institutions.

This study also points that small for gestational age, need for higher supplemental oxygen at 36 weeks of corrected gestational age and presence of pulmonary hypertension are predictors of poor outcomes in our cohort at 2 years of age. Studies done by Mourani et al [21, 22], have shown that pulmonary hypertension may significantly worsen the outcome in BPD infants. Pulmonary hypertension was diagnosed based on echocardiographic findings. Cardiac catheterization to measure pulmonary pressures is not routinely performed in our unit and other NICUs. This may be one of the limiting factors of our study as some cases of pulmonary hypertension might be missed with the use of echocardiography alone [23]. Nevertheless, it remains the best non-invasive screening tool to assess pulmonary hypertension in infants with severe BPD. Even though the accuracy of the echocardiogram may be limited regarding the ability to find a measurable and reproducible tricuspid regurgitant jet velocity to estimate right ventricular systolic pressure, we have an institutional, evidence-based guidelines [24], formed by a multi-specialty committee including neonatology, cardiology and pulmonology teams to diagnose pulmonary hypertension based on echocardiogram criteria in BPD infants. In our study, we found that these above-mentioned risk factors were associated with vent dependency and/or death based on forward step logistic regression analysis. However; only the “sickest” infants with pulmonary diseases were referred to our institution for multi-specialty consultations and were included in this study. We could not include all other infants with less severe BPD in our regression model to make our analysis more robust.

Our institutional data also demonstrates that later gestational age at referral is associated with worse outcomes. Association between the time of referral and poor outcome has not been reported elsewhere. Due to small data set, we are not able to prove any causality. The availability of multi-specialty care at tertiary NICU helps with consistency of care but the evidence for improving outcome secondary to that is lacking. Also, many infants that need tertiary care are already burdened with complex disease problems and delay in transfer to tertiary care NICUs may be secondary to their prolonged sickness or instability of condition precluding early transfer.

Our children’s hospital receives patients from diverse NICUs across 150 mile^2^ area located in 4 different states. The antenatal care, availability of resources, ventilatory strategies, approach to delivery room care, PDA management, nutritional management, level of clinical expertise have been very different among these NICUs which complicated the evaluation and comparison of these infants at admission. NICHD consensus statement for management of BPD infants recommends involvement of interdisciplinary team at 36 weeks of gestation, which was not readily available at many of our referring hospitals. Uniform guidelines for ventilatory, nutrition and infection management in collaboration with multi-specialty teams and interdisciplinary approach may contribute towards improved outcomes [17, 25, 26]. This finding is interesting and may need to be studied further to address this specific question.

RSS at admission was not comparable for these infants due to significant difference of clinical management among referring hospitals. RSS data at the time of tracheostomy placement may suggest severe lung disease in infants, which was also predictive of poor outcome at 24 months of age.

Our long term follow up data at 2 years of age showed that; 66% of infants in our cohort died or were vent dependent. Around 33% of surviving infants had poor neurodevelopmental outcome based on BSID III and GMFCS criteria. The mobility, gross motor and some aspects of neurodevelopmental milestones are dependent on infant’s ventilator dependent status. This may be one of the reasons for such a high number of infants with poor neurodevelopment outcomes in our cohort.

The primary limitation of this study is that the analysis is retrospective in nature, is from a single center and there is no comparison group, hence these data may not be generalizable. Our study is also missing prenatal information to identify the risk factors that may be associated with poor outcomes. Our objective was to identify the risk factors associated with poor outcomes in severe BPD infants; however, as a referral center NICU and due to retrospective nature of our study; we could not collect all prenatal, intrapartum and postnatal characteristics of these infants. This may limit our ability to identify all potential risk factors associated with poor outcomes in severe BPD infants**.** On the other hand, our study represents a largest data till now from a single center in infants with severe BPD requiring tracheostomy.

## Conclusions

In summary, our data identified some clinical risk factors associated with poor outcomes at 2 years of age in single center cohort of infants with severe BPD. These results can be used to assist in counseling parents/families of infants with severe pulmonary disease. Furthermore, clinicians and families need to be diligent towards potential risk for poor neurodevelopmental outcomes of these infants. Modification of these risk factors may be helpful in decreasing severe respiratory and neurodevelopmental outcomes or death. Our data does not identify optimal timing of tracheostomy placement in these babies. Future research directed at understanding this gap will assist referral NICUs in the provision of potentially improved clinical practices to optimize these infants’ health outcomes.

## Data Availability

The datasets used and/or analysed during the current study are available from the corresponding author on reasonable request.

## Abbreviations

BPD: Bronchopulmonary dysplasia
BSID: Bayley’s Scales of Infant Development
GA: Gestational age
GMFCS: Gross motor function classification scale
IQR: Interquartile range
NICHD: National Institute of Child Health and Development
NICU: Neonatal intensive care unit
PDA: Patent Ductus Arteriosus
PHN: Pulmonary Hypertension
PMA: Postmenstrual age
RSS: Respiratory severity score
